# A Study on the Causes and Effects of Stressful Situations in Tourism for Japanese People

**DOI:** 10.3390/bs11110143

**Published:** 2021-10-22

**Authors:** Bình Nghiêm-Phú, Kazuki Shibuya

**Affiliations:** 1 School of Economics and Management, University of Hyogo, 8-2-1 Gakuen Nishimachi, Nishi Ward, Kobe 651-2197, Hyōgo, Japan; 2 College of Tourism, Rikkyo University, 1-2-26 Kitano, Niiza-shi 352-8558, Saitama, Japan; kshibuya@rikkyo.ac.jp

**Keywords:** negative psychological impacts, causes, coping strategies, coping effects, Japan

## Abstract

(1) Numerous studies have been undertaken to investigate the perceived impacts of tourism, particularly from the perspective of local residents. Only a handful have dealt with the coping strategies of this group. In addition, they have largely neglected the successes or failures of the coping strategies and the related consequences. In order to address these gaps, this study aims to investigate the psychological impacts of tourism, focusing on the causes and effects of the negative feelings felt by local residents. (2) Methods: Several qualitative methods, including web archive research, netnography, user-generated content analysis, literature review, and ethnography were employed to gather the necessary data. Japan was selected as the context of this study. (3) Results: This study identified a set of negative feelings and a group of four coping strategies. It also found that the causes of the negative feelings, the bad impacts of tourism, were similar to those in other countries. In addition, the study verified that the effects of the coping strategies were only situational and temporal. (4) Conclusions: Tourism is not stress-free. In order for tourism to sustain, the causes and consequences of its negative impacts must be properly addressed.

## 1. Introduction

As an important industry, tourism can create a lot of positive impacts. For example, tourism can help protect and preserve the natural environment, as well as the cultural and historical heritages of the destinations [1,2]. The industry also brings in more job opportunities and income for local residents, and helps shape or improve development plans of both urban and rural areas [3,4]. In addition, tourism experiences can be considered as stress relievers which help make tourists’ lives happier, and lead to better subjective well-being or quality of life [5]. As a consequence of these positive impacts, local residents may want to further support the development of tourism, while tourists may want to take more holidays [6,7].

However, tourism also is the cause of many negative impacts. For instance, too many tourists arriving in the same short period of time can create unwanted pressures on the infrastructure and sociocultural climate of the destination [8]. The distribution of tourism income may be perceived as unequal by foreign and local stakeholders, or between tourism and non-tourism participants [9]. The perception of these bad impacts may consequently lead to local residents’ negative attitudes toward tourism and its development [10]. Tourism, therefore, is undoubtedly not stress-free [11,12].

Since stresses, tensions, and conflicts can have significant effects on the present and future of tourism development, numerous studies have been undertaken to investigate these issues. For example, much research has examined the negative impacts or stressors from tourism activities, including economic, environmental, sociocultural, medical, and psychological impacts [12,13,14,15,16,17,18]. Several studies have looked at the strategies that employees in the tourism sector use to cope with stressful situations and their causes [19,20,21]. Only a handful have dealt with the coping strategies of local residents [22], and these efforts are mostly quantitative in nature. Thus, the real opinions of the local residents are still somewhat hidden. Given the fact that local residents are the largest population being impacted, the lack of literature on this particular aspect leaves a gap that needs to be addressed. In addition, the earlier studies have largely neglected the successes or failures of the coping strategies employed and the related consequences, such as people’s perceived well-being and attitudes toward tourism. This is another gap in the field of which a better understanding is needed.

The purpose of this study is to investigate the psychological impacts of tourism, focusing on the causes and effects of the negative feelings felt by local residents using qualitative methods. Four research questions (RQ) guide this study.

RQ1. What are the negative psychological impacts (the negative feelings) of tourism as perceived by local residents?
RQ2. What are the causes of negative psychological impacts?
RQ3. What are the strategies that local residents use to cope with negative psychological impacts?
RQ4. What are the effects or outcomes of the coping strategies used?

The context of this study is Japan. In 2019 (prior to COVID-19), nearly 32 million overseas tourists visited this country, while approximately 587 million trips were made by domestic tourists [23,24]. The sheer number of tourists and visitors have undoubtedly put a lot of pressures on the daily lives of local residents at tourist destinations in Japan. Recent academic research, however, mainly focuses on the further exploitation of tourism resources [25,26,27], or on the recovery of disaster-hit areas [28,29,30]. Several studies are trying to advocate and promote more sustainable models of tourism [31,32]. The negative psychological impacts of tourism and related issues, nonetheless, have largely been neglected. Thus, the findings of this study will provide helpful implications for the management of tourism in Japan, as well as to expand the literature on tourism impacts.

## 2. Literature Review

From a psychological perspective, stress can be regarded as a complex phenomenon in which an individual perceives the stressors, experiences the stressful (negative) feelings, and reacts toward the stimuli or situations through certain coping behaviors [33]. Stress can be caused by many daily stressors (e.g., interpersonal communication, living conditions, or workload) or infrequent traumatic events (e.g., death, disease, or disaster) [34]. Consequently, stress can lead to many physical and emotional issues, such as insomnia or anxiety [35,36]. This state can hinder the normal performance of daily activities, such as driving or studying [37,38]. Long-term stress can significantly reduce the well-being and longevity of individuals [39]. Too much stress, as the result of perceived negative impacts, may potentially diminish local residents’ support for the further development of tourism in the future [13,40,41].

In the tourism sector, many factors can be considered as the causes of local residents’ stresses (in other words, the negative psychological impacts of tourism). For example, in Jamaica, the problems are very diverse, ranging from infrastructural and environmental issues (e.g., traffic congestion, changed traffic patterns, pollution, and overused facilities) to economic and social issues (e.g., no tourists, no jobs, no benefits, no money, increased cost of living, and police harassment) [15]. In Korea, the traffic problems, security risks, and economic costs seem to be more prominent [17]. In the Solomon Islands, the local residents are irritated by the possibilities of diminishing social capital, foreign influence, and economic development [42]. Thus, the perceived stressors obviously differ among destinations and the specific types of tourism activities at the destinations.

Fortunately, there are certain ways for local residents to reduce the impacts of tourism-induced stressors. According to Jordan et al. [22], four strategies (problem-focused, social support, positive outlook, and wishful thinking) can be adopted. Specifically, with the problem-focused method, individuals can choose to talk to other people about the problems, or fight for what they want. With the social support method, individuals may seek advice or accept sympathy and understanding from other people. With the remaining two strategies, which are more intrinsic than the former extrinsic ones, all they have to do is create and maintain a positive attitude toward life and the uncomfortable situation.

In addition, stress-coping strategies can also be found in the experiences of tourism-related employees. For example, hotel employees may participate in leisure activities, which are extrinsic, to reduce stress [21,43]. If the employees’ attitude toward leisure is adaptation, their perceived well-being can be improved. However, if their attitude is avoidance, the outcome will be the opposite. On the other hand, hotel employees may simply adopt an intrinsic method, such as keeping their hopes up, and ensuring that their tasks are properly performed [44].

## 3. Methods

**Figure 1:**
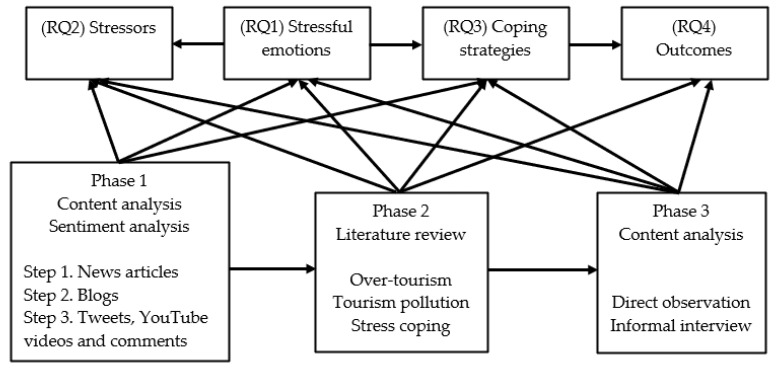
Research process and materials.

Given the exploratory nature of this study, several qualitative methods, including web archive research, netnography, user-generated content research, literature review, and ethnography [45,46,47,48,49,50] were employed to gather the necessary data (Figure 1). The analysis of the data was mainly guided by the content analysis and sentiment analysis methods [51,52].

### 3.1. The First Phase of Data Collection and Analysis

In the first phase of the data collection and analysis process, the researchers did archival and netnography research to obtain context-based data [45,49,50]. In addition, as Japan was selected as the setting of the study, only information about Japan was collected (context-based data), and Japanese was the language of the search. This phase included three steps.

#### 3.1.1. The First Step of Data Collection

The first step involved an archival research [45] on news articles published by local agencies. Generally, this type of data helps reveal public opinions from the perspectives of a third party—the journalists [53,54]—which is an important reference before encountering more in-depth information contributed by the local residents themselves.

In this study, the contents of the news articles, which were kept in online databases of the news agencies, were treated as the original archives from which information about tourism impacts in Japan in recent years could be extracted. Two specific databases available and accessible to both researchers were employed, including one of a national newspaper (*Nikkei*) and one of two regional newspapers that produce news for two important areas of Japan (*Chunichi*, for Nagoya, and *Tokyo*, for Tokyo). The use of two types of database differed from previous studies which also examined Japanese newspapers [53,54] in that it did not limit the information to a large or national scale but also took into account information from smaller regions to obtain more diverse opinions and observations.

As a result of this practice, 26 newspaper articles published over a 10-year period (2011–2020) were identified with the help of two general keywords: “tourist destination” and “local residents.” This time-frame was considered because it covered the long-term yet updated information about tourism impacts in Japan historically and chronologically, which is a necessary condition to guarantee the validity of the data [55]. The contents of the articles were kept in an Excel file. This step was conducted in September and October 2020.

#### 3.1.2. The Second Step of Data Collection

The second step concerned a user-generated content research [50] on the blogs written by Japanese individuals. Generally, blogs can provide relevant insights into the local residents’ opinions from their own perspectives or from these of the people who have proper knowledge about them (e.g., academic researchers) [56,57]. In this study, the contents of the blogs were also referred to diverse the understanding about tourism impacts in Japan.

Google was used as the search engine in this step, and again, “tourist destination” and “local residents” were employed as the keywords. Seven blogs were found (2017–2020). The keywords were used as the first-level filter [58] and a critical and selective reading of the researchers served as the second-level filter [59]. The contents of the blogs were also kept in an Excel file. This step was undertaken in October and November 2020.

#### 3.1.3. The Third Step of Data Collection

The third step was a netnography research [49] undertaken to collect the content directly generated by local residents in Japan about the impacts of tourism in their country [50]. Overall, netnography can be regarded as the application of ethnography on the Internet. The netnographic methods may range from pure observation to full participation in online communities to gather the necessary data about such communities. Interviews and discussions may or may not be included in these processes. In this study, to avoid stresses unnecessarily and unintentionally imposed on the subjects and to maintain the neutral position of the researchers, non-participant observation was employed as the main netnographic method [60]. Twitter and YouTube, two of the most popular video sharing platforms and social media sites in Japan [61], were selected as the main sites for observations.

Specifically, the researchers used the two general keywords of “tourist destination” and “local residents” to identify the relevant YouTube videos and Twitter threads that contained contents about tourism impacts in Japan. The YouTube video watchers and commentors, and the tweet posters and followers, were treated as two communities into which netnographic observations were implemented.

In the beginning (October 2020), the researchers only watched the videos and read the comments and tweets to get familiar with the contents and their contributors. Each observation session might last one to two hours. During this period, the researchers realized that the negative feelings were often specifically connected with over-tourism or tourism pollution. Therefore, the research group decided to replace the keyword “tourist destination” by “over-tourism” and “tourism pollution” in the search and observation of other contents and communities. The relevant contents (YouTube video comments and tweets) contributed between January 2018 (when the first related tweet was found) and October 2020 were saved in an Excel file for later use. Overall, YouTube comments and tweets have been employed as a reliable source of information to understand both the sentiments and opinions of the general public [62,63,64,65,66], which are relevant for the purposes of this study.

Next (November 2020–March 2021), the researchers conducted non-participation observation of the communities identified through the new keywords (over tourism, tourism pollution, and local residents) on a bi-weekly basis. The purpose was to detect the new contents (videos, comments, and tweets), the rhythm and frequency of the postings, and the incidents that triggered the new contents or postings. Each session lasted 30 to 60 min. At the end of each session, the researchers saved the new contents in the existing Excel file. At the end of the netnographic observation period, a total of eight YouTube videos and 70 comments, and 121 tweets, directly involved the topics of this study, were collected. The narration of the reporters and the talk of the interviewees in the YouTube videos were also analyzed, since they could provide alternative references. However, news articles, which served as the rousers of some tweets at certain times, were overlooked to avoid redundancy (items describing an issue already mentioned by the materials gathered in the first or second steps) and irrelevance (items describing issues in countries other than Japan).

#### 3.1.4. The Analysis of the First Phrase’s Data

After the Internet-based data were compiled (Table 1), the researchers analyzed them using the sentiment and content analysis methods [51,52]. Specifically, the researchers sought the adjectives which helped reveal the negative feelings (RQ1) perceived by local residents as the consequence of tourism activities at their respective destinations (sentiment analysis) [52]. After that, the researchers traced the causes of (RQ2) and the strategies (RQ3) used to cope with these feelings in the text, if there were any, with the help of the content analysis technique [51].

The observations of each researcher of the feelings, the causes, and the strategies were entered in an Excel file for individual mastering. After that, these two sets of data were combined to generate the answers to the research questions. The researchers went through the results together to identify the differences or errors, if there were any. Total agreement was reached before concluding this procedure to ensure the reliability criterion of the data analysis [55].

### 3.2. The Second and Third Phases of Data Collection and Analysis

In the second phase, the data collected in the first phase was compared with the findings of previous studies about over-tourism, tourism pollution, and stress coping to find the differences and similarities between the situations in Japan and other countries, and between the situations in tourism and other business sectors. This was done to check whether the data could reliably represent the opinions and sentiments of the local residents or not (data triangulation) [67]. The review of the literature about stress coping can further suggest the potential effects and outcomes of the coping strategies (RQ4).

In the third phase, the researchers conducted ethnographic observations [47] at the areas often mentioned by the contributors in the first phase to further validate the information (October 2020–May 2021). The sites include Kamakura (south of Tokyo) and Kyoto, two cities where actual practices were being implemented to reduce the impacts of tourism on local residents. When and where possible (Kyoto), the researchers informally interviewed local residents (*n* = 13) to deepen the understandings of the coping strategies (RQ3) and their effects and outcomes (RQ4). To reduce the stresses for the participants and the surrounding people, the interviews were informally implemented. The researchers did not record the interviews but noted the major information in a journal for later use. In addition, pictures, as evidence of the causes of the negative feelings (RQ2), were also taken for the same purpose. The fieldwork was concluded when no new information was being retrieved, at the point data saturation was reached [68]. The notes and the pictures were later analyzed (content analysis) [51] to strengthen the answers for the research questions.

### 3.3. Limitations and Merits of the Methods

The data collection methods have two major limitations. First, although the information was about the local residents, it was not necessarily contributed by local residents. In other words, it can be assumed that at least some of the data collected probably came from people who were not residents of the locality. Second, opinions of the local residents which were not heard or expressed were unavoidably neglected. As a consequence, the findings of this study may not perfectly represent the residents’ feelings, thoughts, and opinions, despite the triangulation effort.

These methods, on the other hand, have several merits. First, since the data collection was principally undertaken in the last four months of 2020 and the first five months of 2021, these methods helped reduce the threat of COVID-19 that the researchers might have been exposed to [69,70]. They also helped trace the information to earlier years when COVID-19 was not a threat to tourism activities and to the daily lives of local residents. Second, these methods helped the researchers to focus on areas where tourism really has a negative psychological impact on the local people. The efforts and resources, therefore, could be maximized.

## 4. Results and Discussions

### 4.1. Negative Feelings and Their Causes

In recent years, local residents at tourist destinations in Japan have been experiencing a lot of negative feelings, ranging from milder ones, such as nostalgia and sadness, to hasher ones, such as anger and frustration (Table 2). Thus, the negative feelings observed in Japan are very diverse and context-specific, making the situation different from what the theories and other settings indicate [12,15,16,22,71,72]. However, if causes of the negative feelings are not dealt with properly, they will lead to more severe symptoms of stress, anxiety, and depression [35,36], which will affect the overall well-being of the residents ever more greatly [39]. For example, a person wrote in their YouTube comment that they felt “exhausted” and “annoyed” when they were told by a young male tourist not to carry their baby carriage, or when they were scolded by a foreign tourist because their baby was restless in a crowded bus. Another person mentioned in their YouTube comment that they were so “frustrated” that they wanted to murder the tourists because of their behaviors.

There are a variety of causes of the overall negative feeling (as opposite to the overall positive feeling). From an economic perspective, tourism development has increased the price of real estate at the destinations, which consequently leads to difficulties in buying or renting an apartment or an office. Public transportation, parking areas, and daily life goods are also affected similarly. The unfair distribution of income from tourism is another cause of the negative feelings. Examples of this particular cause are numerous. One person observed that all the shops were full and all the convenience store lunch boxes were sold out at noon (YouTube comment). Consequently, nearby local residents could not buy what they needed there and then. Another person wrote that they could not find a spot in a local parking lot, and that the cost was high (YouTube comment). In other words, local residents might have to go further and pay more for a parking lot. Alternatively, an individual in Miyakojima (Okinawa), a small island with a population of only 53,000 people but hosting 600,000–700,000 visitors a year, claimed in their tweet that the locals could not even take a taxi when a cruise ship arrived. They could also not go to the local beaches, or make much money from these tourists.

From a social standpoint, the arrival of large numbers of tourists have caused a lot of pressures on the general infrastructure of the destinations (e.g., roads, public transportation, parking areas, hospitals, restaurants, convenience stores, and water supply), and reduced the access to these goods and services for local residents. The development of for-tourism facilities also adds further burdens. For example, a blogger in Nagoya said that they once saw a big tourist bus stopped right at the busiest part of the parking area of a department store which might cause accidents. Another blogger noticed that the noise made by guests at “minpaku” (Airbnb-type private accommodations) had deteriorated local residents’ living environment. These impacts are not insignificant.

Through a cultural lens, tourists, especially foreigners who do not understand or comply with the culture and traditions of the destinations, can create unwanted irritations and troubles for local residents. Examples include trespassing on private property, stalking local figures (such as Maiko girls in Kyoto), unpermitted photo taking, uncivilized use of toilet facilities, not following eating and smoking manners (eating and smoking while walking), having loud conversations, sitting in sacred and off-limits or closed areas, and touching of objects and products that are not permitted, among others. In addition, the development of for-tourism activities and businesses has also spoiled the identities of many of these destinations. For example, one individual noticed in their tweet that shops that originally existed in Kyoto have disappeared, and the number of shops disguised as Kyoto shops began to increase, and the wrong depiction of Kyoto was advertised.

**Figure 2:**
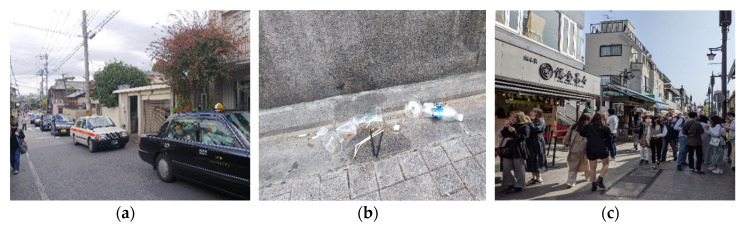
Examples of visitors’ actions (Source: Authors) (**a**) Kyoto—22 November 2020, (**b**) Kyoto—3 October 2020, (**c**) Kamakura—31 March 2021.

From an environmental point of view, the congestion and overcrowded-ness have led to air, noise, and visual pollution at tourist destinations (Figure 2a). In addition, litter and trash could be noticed everywhere, even in large quantities, around trash bins and garbage collection areas (Figure 2b). Finally, from a medical viewpoint, tourists visiting destinations during the COVID-19 pandemic might cause potential health risks for the residents (Figure 2c).

The abovementioned causes of local residents’ negative feelings, undoubtedly, are parts of the negative impacts of tourism. These causes, thus, are not only observed in Japan, but also in other countries [12,15,17,42]. The negative impacts, nonetheless, seem to be more severe when a large number of tourists, both domestic and foreign, continually visit the destinations. In addition, the problems are perceived as more serious where tourist sites and residential areas are too close to one another. This phenomenon is regarded as over-tourism [73] and is the main cause of tourism pollution in Japan.

### 4.2. Coping Strategies and Their Effects

As Japan is a very stressful society, many studies have examined strategies that Japanese people use to cope with personal stress. For example, Nagase et al. [74] reported three strategies: avoidance, problem-focused, and emotion-focused. Alternatively, Kato [75] mentioned three strategies: distancing, reassessing, and constructive coping. In a more recent study, Otsuka et al. [76] listed other strategies, including “giving up on problem solving”, “enduring problems patiently”, “smoking”, “drinking alcohol”, “exercising”, “enjoying hobbies”, and “sharing worries”.

It is observed in this study that local residents in tourist destinations in Japan have been using some of these strategies to cope with the negative feelings caused by the external impacts of tourism. First, many locals have opted to simply get used to (慣れる) and put up with (我慢する) the situation. Japanese people, interestingly, are very good at being this kind of patient [77]. In such cases, no further actions are taken.

Second, they might choose to voice their concerns (speak out), such as reporting the problems to administrators, consulting with the police, or writing comments on blogs, Twitter, or YouTube. Some individuals even suggested and recommended strategies to deal with the negative impacts. For example, an individual thought that Kyoto could learn from the lessons of museums in Europe, where they restricted admission of visitors by prior reservations (YouTube comment). It was, therefore, possible to limit the number of tourists that could visit Kyoto in a day.

**Figure 3:**
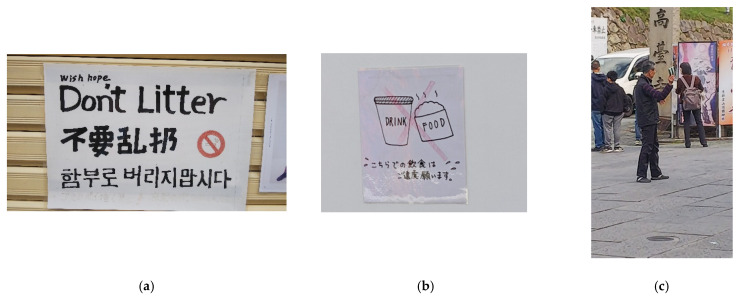
Examples of local residents’ actions (Source: Authors) (**a**) Kyoto—3 October 2020, (**b**) Kamakura—31 March 2021, (**c**) Kyoto—20 November 2020.

Third, some locals prefer taking action, including finding alternative modes of transportation (walking or cycling), setting up notice boards (Figure 3a,b), collecting trash, or participating in the activities of residential organizations, such as giving out pamphlets, directing traffic, and guiding tourists (Figure 3c). These activities have been seen in, for example, Kamakura, Kyoto, and Okinawa.

Fourth, a minority of people adopted the distancing strategy by closing their business or moving out of the destination. The reasons, however, might not solely be negative tourism impacts. A person, for example, wrote in their YouTube comment that they were once a citizen of Kyoto but relocated to Hida Takayama (Gifu prefecture). However, they also hoped that their new place would not become a second Kyoto or they would have to move again.

These strategies differed from those surveyed in Jamaica in an earlier study [22]. Perhaps this is due to the difference in data collection methods, where the latter used a predetermined scale to examine local residents’ opinions. The strategies also differed from those adopted by tourism employees [21,43,44]. The reason is that tourism impacts are somewhat personal for tourism employees’ jobs and lives, while they are both personal and social issues for the local residents.

The effects of the four coping strategies (getting used to/putting up with, speaking out/voicing, taking action, and distancing) vary, according to the literature [74,76]. Specifically, avoidance (getting used to/putting up with) can hardly reduce the stress but can significantly increase the level of depression. The effects, however, are reversed when using problem-focused strategies (speaking out/voicing, taking action, and distancing). Yet, they are not long-lasting since the causes of the negative feelings, the bad impacts of tourism, are not properly dealt with. In other words, the coping strategies are only situational and their effects are only temporal.

This speculation is supported by all types of data: Internet-based materials, onsite direct observations, and informal interviews. Specifically, the economic, social, cultural, environmental, and medical impacts are permanently attached to tourism activities [12,13,14,15,16,17]. Where there is tourism, there are impacts. As Japan is aiming to continually increase its inbound tourism [78], voices of distress, complaints, accusations, and even hatred can be heard everywhere. Even when Japan closed its border to international tourists as a safety measure, for example, because of COVID-19, large waves of domestic tourists could not be stopped, except when the first national emergency was declared (7 April–25 May 2020) [79]. Thus, until proper strategies are initiated and undertaken by central and local governments, residents at tourist destinations in Japan have to constantly live with the negative feelings caused by the bad impacts of tourism.

## 5. Implications of the Results

### 5.1. Theoretical Implications

Overall, Japan is a developed economy. However, in the tourism industry, Japan still is a developing destination. Thus, the negative impacts of tourism in Japan, in general, and the consequent negative feelings felt by local residents, in particular, are similar to these observed in other countries around the world. However, the strategies that local residents are using to cope with these impacts have a direct connection with Japan and its developing context. For example, the four coping strategies (getting used to/putting up with, speaking out/voicing, taking action, and distancing) distinctively reflect the values of Japanese people and its culture (harmony, empathy, and patience) [77]. In addition, they maintain the “omotenashi” (sense of hospitality) philosophy of Japanese business [80]. These elements may have restrained the local people’s reactions to the rapid increase of tourism in Japan. In addition, they also result in lower levels of tourismphobia than might have been expected [81,82]. Serious consequences, such as protests and anti-tourism movements [83,84], have not yet developed.

### 5.2. Practical Implications

In order to sustain the long-term development of tourism in Japan, Japanese central and local governments, as well as tourism businesses in the country, may consider the following directions. First, the capacity of each tourist destination [85] should be carefully calculated and ensured. Here, the approach of the domestic agriculture industry, in which quality is valued more than quantity [86], could be referred to. Second, the positive contributions of tourism [87], especially those to the national and local society, culture, economy, and environment, should be better conveyed to the public, including local residents at tourist destinations. Without these to counter the negative impacts, support from local residents cannot be sustainably achieved.

## 6. Conclusions

The purpose of this study was to investigate the psychological impacts of tourism in Japan. Using a variety of qualitative methods to collect and analyze the data, this study identified a set of negative feelings and a group of four coping strategies that are specific to the Japan context. It also found that the causes of the negative feelings, the bad impacts of tourism, are similar to those in other countries. In addition, the study verified that the effects of the coping strategies the Japanese people are using are only situational and temporal. As a consequence, recommendations to sustain the development of tourism in Japan were proposed.

The results of this study point to encouraging directions for Japan’s tourism industry to follow. However, to address the limitations of the databases, future studies should expand the extent and load of fieldwork in order to collect additional information from the affected residents and those who have not been heard from yet. Moreover, future studies should consider several issues unexamined or only briefly observed in this study. For example, are perceptions of tourism impacts similar or different among local residents, tourism administrators, businesses, and tourists? How do the negative feelings affect local residents’ attitudes toward the tourism administrators, businesses, tourists, and the place? Answers obtained through these attempts will further deepen the understanding of tourism impacts, including psychological impacts, as well as provide more helpful implications for the management of sustainable tourism in Japan and other countries.

## Figures and Tables

**Table 1:** Internet-based data.

Types of Data	Number
News articles	26
Blogs	7
YouTube videos (comments)	8 (70)
Tweets	121

**Table 2:** Examples of negative feelings.

Original in Japanese	Translation in English
憤りを感じている	Indignant
痛みを感じている	Pain
イライラしている	Frustrated
苛立っている	Frustrated
うんざりしている	Fed up, tired
怒っている	Angry
悲しい	Sad
苦悩している	Distressed
苦しめられる	Distressed
怖い	Scared, fleftened
ストレス	Stressed
心配している	Concerned, worried
疲れている	Tired
辛い	Bitter
懐かしい	Nostalgic
疲弊している	Exhausted
不安	Anxious, insecure
不平不満	Discontented
不満	Dissatisfied, discontented
辟易している	Tired, overwhelmed

## Data Availability

The data used for this paper are available upon request from the corresponding author.
